# Interpretable evaluation for the Brunnstrom recovery stage of the lower limb based on wearable sensors

**DOI:** 10.3389/fninf.2022.1006494

**Published:** 2022-09-08

**Authors:** Xiang Chen, DongXia Hu, RuiQi Zhang, ZeWei Pan, Yan Chen, Longhan Xie, Jun Luo, YiWen Zhu

**Affiliations:** ^1^Department of Rehabilitation Medicine, The Second Affiliated Hospital of Nanchang University, Nanchang, China; ^2^Fuzhou Medical College, Nanchang University, Nanchang, China; ^3^Shien-Ming Wu School of Intelligent Engineering, South China University of Technology, Guangzhou, China

**Keywords:** rehabilitation evaluation, Brunnstrom recovery stage, wearable sensor, machine learning, feature importance

## Abstract

With the increasing number of stroke patients, there is an urgent need for an accessible, scientific, and reliable evaluation method for stroke rehabilitation. Although many rehabilitation stage evaluation methods based on the wearable sensors and machine learning algorithm have been developed, the interpretable evaluation of the Brunnstrom recovery stage of the lower limb (BRS-L) is still lacking. The paper propose an interpretable BRS-L evaluation method based on wearable sensors. We collected lower limb motion data and plantar pressure data of 20 hemiplegic patients and 10 healthy individuals using seven Inertial Measurement Units (IMUs) and two plantar pressure insoles. Then we extracted gait features from the motion data and pressure data. By using feature selection based on feature importance, we improved the interpretability of the machine learning-based evaluation method. Several machine learning models are evaluated on the dataset, the results show that k-Nearest Neighbor has the best prediction performance and achieves 94.2% accuracy with an input of 18 features. Our method provides a feasible solution for precise rehabilitation and home-based rehabilitation of hemiplegic patients.

## Introduction

Stroke is an acute cerebrovascular disease caused by bleeding or blockage of blood vessels in the brain. Deaths from stroke account for 11% of all deaths in the world and rank second among the leading causes of death (World Health Organization, 2020). In some regions, such as Bulgaria, 3 out of 1,000 deaths are due to stroke (Kim et al., 2020), indicating the high incidence and mortality of stroke. 80% of stroke survivors exhibit hemiplegia due to loss of central nervous system control of the motor system, resulting in abnormal coordination of the patient’s muscle groups and abnormal muscle tones (Qiu et al., 2018), such as pain, swelling, fatigue, and coordination problems (Alonso-Vázquez et al., 2009), which severely affects the patient’s rehabilitation exercises and quality of life, and the risk of falls is raised. Therefore, rehabilitation evaluation and treatment of stroke has become a major issue in public health, and hemiplegic gait analysis has become an important part of rehabilitation.

Studies have shown that timely, active, and accurate rehabilitation interventions can restore self-care to most patients with hemiplegia and performing accurate and appropriate rehabilitation is promising for the function of the lower limb (Nepveu et al., 2017; Li et al., 2018; Hosseini et al., 2019). Berengueres et al. (2014) found that if muscles are not actively exercised when the foot is fixed in corrective shape, the muscles are not activated by training, which can make protective shock-absorbing function, effective gait and other complex functions to be impaired. A proper hemiplegia evaluation is crucial for correct treatment plan for hemiplegia to better restore the patients’ muscle function. However, current clinical evaluation is based on clinician observation and clinical scale evaluation, and the evaluation of stroke severity is based on the patient’s medical history as well as the examination.

The Brunnstrom recovery stage (BRS) is one of the most popular motor function evaluation methods, which consists of three items for the arm, hand, and lower limb, each with six levels of flaccidity, spasticity, co-movement, partial dissociative movement, dissociative movement, and normal (Brunnstrom, 1966), as shown in Table 1. Due to its high correlation with motor recovery in stroke patients, BRS has been extensively used in clinical as well as scientific research (Huang et al., 2016). However, the results of the observation method rely heavily on the observer’s level of observation skills and clinical experience. Besides, the evaluation process has a great impact on the patient’s comfort level, and the tedious operation also tends to cause physical and mental fatigue and discomfort, which cannot be recorded in real-time (Zhao et al., 2017). Therefore, the observation method is mostly used for the comparison of patients’ stages of rehabilitation, which cannot meet the requirements of clinical applications (Tran et al., 2018).

**TABLE 1:** Description of each Brunnstrom recovery stage (Naghdi et al., 2010).

Stage	Description
Stage 1	Lack of movement in the extremities.
Stage 2	Slight voluntary motor response in the extremities and the onset of spasticity.
Stage 3	Patients can control synkinesis autonomously, spasticity is severe.
Stage 4	Patients have control of detachment movements and spasticity begins to diminish.
Stage 5	The diminished role of co-movement and enhanced control of separate movement.
Stage 6	Normalization of movement and disappearance of spasticity.

To address the shortcomings of the observational scale method, devices such as visual monitoring systems and plantar pressure monitoring systems have been used in more advanced rehabilitation units and laboratories for hemiplegic gait analysis. However, the visual system and the dynamometric platform are complicated to operate, the testing process can only move within a certain area, it does not facilitate the timely adjustment of the treatment method, and it also causes privacy issues under the surveillance of cameras. Therefore, the above devices still do not meet the conditions for community-based rehabilitation, i.e., they do not allow for immediate evaluation and feedback of the patient’s gait problems, and the patient still needs to travel between home and hospital with high frequency, which is a burden for patients with hemiplegia who are already having difficulty walking. Fortunately, the research conducted with the above devices provides a solid practical basis for the clinical application of wearable sensors, such as the feasibility of evaluation methods based on joint angle and plantar pressure.

In recent years, wearable sensors have highlighted great potential for clinical evaluation (Stuart et al., 2022; Zhang et al., 2022). Berengueres et al. (2014) formed a smart insole by means of pressure-sensitive sensors placed in the mid-lateral aspect of the insole to monitor the pressure in real-time, use pressure thresholds to detect excessive internal rotation and pronation of the foot and provide feedback to the user to indicate if an abnormality is occurring. The Smart Textile Sock integrates five pressure sensors and utilizes a pressure vector algorithm for pre-and post-rotation detection. It is relatively low cost and can be used both indoors and outdoors (Domínguez-Morales et al., 2019). In addition, diagnosis can be assisted by a physician using an Inertial Measurement Unit (IMU) mounted at the heel by calculating parameters such as gait speed and gait (Qiu et al., 2018). With the development of machine learning, deep neural network analysis of the data from the IMU installed at the calf has been performed to identify drop-foot gait, pirouette gait, hip hiking gait, and rear knee stroke gait (Wang et al., 2021). These methods proved to be effective using wearable sensors to assess the hemiplegic gait at a relatively low cost. However, this part of the study could only detect the presence or absence of hemiplegic gait or abnormal gait type and could not quantify the degree, making it difficult to obtain a definite severity of hemiplegia (Mannini et al., 2016; Hsu et al., 2018). On the other hand, most studies have focused on the association of upper limb behavior with the evaluation of FMA and ADLs, and few studies have focused on the degree of lower limb function based on the analysis of gait parameters. Only 7 of 34 papers related to wearable sensors and machine learning mentioned the lower limb and 3 mentioned gait (Boukhennoufa et al., 2022). In general, studies have focused on the recognition of daily movements, motion classification, and clinical evaluation, and further research is needed on the use of wearable sensors for gait analysis for clinical evaluation. In particular, medical professionals are interested in the contextual information of the evaluation results, i.e., the interpretability of the evaluation algorithms (Capela et al., 2015).

According to the surveyed data, few scholars have focused on feature importance-based lower limb functional evaluation, and few have seen a combination of wearable sensors based on seven wearable IMU as well as dynamometric insoles, or feature analysis of lower limb functional evaluation. By using dual-source data fusion consisting of IMU and plantar pressure, the features extracted in the present paper are more accurate and also form a variety of multiple pre-selected features, including kinematic parameters, plantar pressure parameters, and spatial parameters. Berengueres et al. (2014) indirectly determined inversion and eversion by monitoring plantar pressure with sensors placed on the lateral side of the midfoot insole, but neither gave an evaluation of the degree of lower limb function. Also, some studies have used deep learning neural networks to increase the accuracy of the evaluation (Boukhennoufa et al., 2021), but this failed to provide more contextual information to the physician for this evaluation method.

The focus of this study is on using comfortable wearable sensors for intelligent, self-service and reliable evaluation, to achieve comprehensive, home-based and immediate rehabilitation. In this study, we use the joint motion of the lower limb and the distribution of plantar pressure to assess the movement status of stroke patients. Considering that stroke patients generally need to undergo lower limb rehabilitation, this study objectively assesses the BRS-L in a graded manner. Due to the convenience of the monitoring method in this study, the therapist can dynamically adjust the treatment plan during the prime rehabilitation period of the hemiplegic patient, which is beneficial for the patient’s recovery. One benefit that distinguishes this study from other methods is that our method is interpretable, focusing on the selection of gait parameters and characteristics.

The research methodology is first introduced in sections “Experimental protocols” and “Materials and methods,” including the design and conduct of experiments, equipment introduction, data preprocessing, calculation, analysis and selection of parameters and features, and model construction and performance evaluation. Section “Results,” this paper presents the results of the experiments, which are finally discussed in section “Discussion.”

## Experimental protocols

We recruited volunteers to collect their gait data using wearable IMUs and plantar pressure insoles, and the following is the experiment setup.

### Participants

The following criteria were utilized to recruit participants: (1) Unilateral hemiplegia and undergoing rehabilitation in hospital; (2) Age between 18 and 80 years; (3) Brunnstrom recovery stages of lower limb (BRS-L) III-V; (4) Normal mental status and consciousness; (5) Subject can walk 10 meters indoors (with or without assistive devices). Because patients in the stages of BRS-L I-II are unable to walk (independently or assisted), we excluded them. To increase the sample size of the data set, we also recruited 10 healthy individuals.

Before carrying out this experiment, we obtained approval of the Ethics Committee of the Second Hospital of NanChang University. Voluntary subjects signed informed consents before the experiment. Finally, 30 individuals participated in the experiment, including 20 stroke subjects (aged 57.7 ± 8.7 years, with a height of 164.5 ± 6.9 cm and a weight of 61.5 ± 9.1 kg) and 10 healthy subjects (aged 34.3 ± 2.5 years, with a height of 173.6 ± 4.6 cm and a weight of 63.2 ± 6.6 kg).

### Experimental setup

**FIGURE 1:**
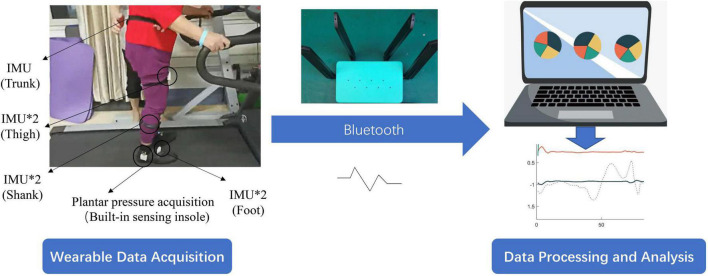
Schematic diagram of wearable sensing and data analysis device.

Figure 1 shows the experimental setup. Seven IMUs were selected for inclusion in the experiment and were strapped to the patient’s bilateral feet, bilateral calves, bilateral thighs, and waist. The selected IMU sensor uses a JY901s inertial sensor chip module made by Wit Motion, which includes an accelerometer, magnetometer, and gyroscope. Supplementary Table 1 displays the chip’s specific parameters.

In addition, a smart insole was added to the shoe to obtain plantar pressure. We designed and manufactured the smart insole, which incorporates 8 pressure sensors. The position arrangement of pressure sensors is derived by minimizing the pressure center position measurement error. Please see article (Xian et al., 2021) for further details. The sampling frequency of IMU and pressure sensor is 200 Hz. The above devices use Bluetooth wireless transmission. To achieve multi-sensor synchronization, the upper computer examines the data transmitted by the sensor at a frequency of 200 Hz and considers the most recent frame of data received by each sensor as the data of the current moment.

After the devices are worn, the IMUs need to be calibrated to obtain stable posture data. All patients first walked at their comfortable walking speed in the shoes with built-in sensory insoles to perform an adaptation familiarization test. After finding their comfortable walking speed and reaching a stable gait, they begin to walk formally for about 2 min while recording plantar pressure data. The experiment is conducted in a quiet environment in a hospital.

## Materials and methods

**FIGURE 2:**
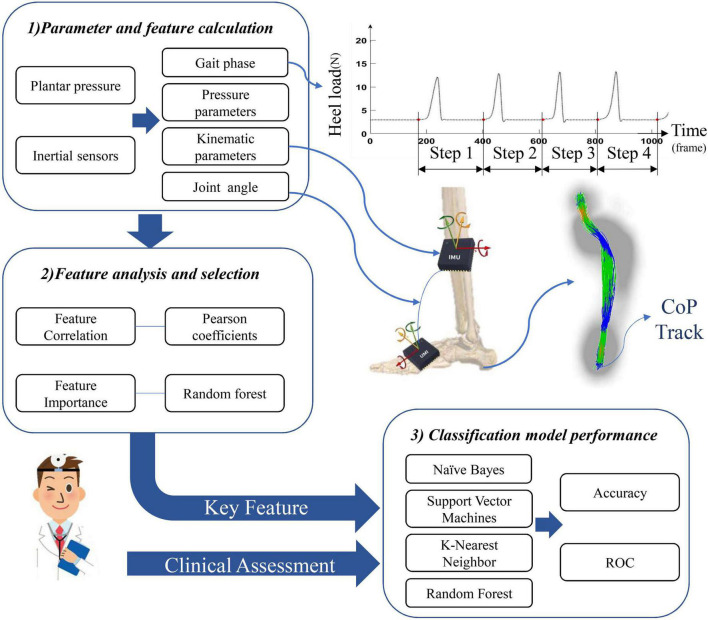
Flowchart of the data processing.

The method designed in this paper is shown in Figure 2. The collected IMU data and plantar pressure data are used for further parameter and feature calculations. First, the data are filtered and divided into steps. Then, the feature data of each step are calculated and correlation and importance analyses are performed for each feature. Finally, the filtered features are used for model training and realistic lower limb function evaluation.

### Data preprocessing

To obtain a well-organized dataset, the collected raw data needs to go through a series of preprocessing.

#### Low-pass filtering

To eliminate the noise data, a 6-order Butterworth low-pass filter with a cut-off frequency of 15 Hz was used to preprocess the data.

#### Gait cycle segmentation

A gait cycle consists of heel touch, mid-foot touch, heel off, forefoot touch, forefoot off and swing. Since normal gait generally begins with heel touch, this study uses the response of heel sensors for touch judgment. We consider the sudden increase in the value of the pressure sensor under the heel as the activation signal, in other words, as the beginning of current gait cycle and the end of previous gait cycle.

#### Gait phase segmentation

Because hemiplegic patients do not have multiple support subphases as normal due to the muscle weakness, we only divide gait cycle into the double support phase and single support phase, i.e., the affected side touches the ground as the affected side double support phase, and then enters the affected side’s single support phase until the healthy side’s heel touches the ground.

### Feature extraction

After the preprocessing, gait features are extracted from the gait cycles. As indicated in Table 2, the gait features include primarily spatial parameters and their characteristics, temporal parameters and their characteristics, and plantar pressure parameters and their characteristics.

**TABLE 2:** Descriptions of gait features.

Gait feature	Description
Gait line	The trajectory line formed by the position center of pressure
Regional pressure	Pressure ratios at different locations on the plantar
Gait phase	The proportion of each phase of a gait cycle
Acceleration	Three-axis acceleration based on sensor coordinate system
Step length	Length of each step forward
Joint angle	The rotation angle of the joint during the movement

After the above rotation matrix ${}_{f}^{l}\,R$, the quadratic numbers can be transformed into Euler angles, which are finally translated into plantarflexion and dorsiflexion, inversion and valgus, and internal and external rotation angles of the ankle joint. Besides, this paper uses the quadratic integration of acceleration for the calculation of gait length during walking. The average velocity of the human walking process during the gait cycle can be obtained with the gait cycle time length. The special feature of this paper is that the moment of touching the ground and leaving the ground judged by the plantar pressure can be more accurate to the integration start time of the stride length so that the stride length data can be obtained more accurately. And the calculation process of quadratic integration is as follows:

At moment *t*, the transformation matrix of the attitude of the foot sensor relative to the Earth coordinate system is Rtfe, and the three-axis acceleration of the foot sensor relative to the sensor coordinate system output at this time is **A***cc*_**f***t*_. Since the accelerometer is affected by the acceleration of gravity at this moment, the actual acceleration of motion during the walk **A***cc*_**f***rt*_ needs to be subtracted from the acceleration of gravity **G**.


(1) A⁢c⁢cf⁢r⁢t=A⁢c⁢cf⁢t-RtTfe⋅G

Since the Y-axis direction of the sensor is basically the same as the travel direction when the foot sensor is arranged, the Y-axis direction of the sensor motion acceleration A⁢c⁢cf⁢r⁢tY can be obtained after the quadratic integration of the time from the *t_1_* moment after the toe leaves the ground to the *t_2_* moment when the heel on the same side hits the ground, corresponding to the step length *l*:


(2) l=∬t1t2A⁢c⁢cf⁢r⁢tY⁢dt

The features shown in Table 2 can be classified as follows: for the gait phase parameters, the percentage of each gait phase can be reflected as one of the features for gait. For spatial parameters, ankle mobility and mean stride length are also commonly used gait parameters. For plantar pressure, the ratio of pressure in each plantar region is a feature of interest. According to plantar anatomy, the plantar can be divided into three major regions: forefoot, midfoot, and hindfoot, and the data are shown by the sensors located in the corresponding regions in the smart insole can be summed up to obtain the total pressure of the plantar regions and the regional pressure ratio can be derived. In addition, the trajectory of the plantar center of pressure has been shown to reflect a variety of gait characteristics. The Equation for calculating the center of plantar pressure is shown below:


(3) $$\begin{array}{c} X_{c\,e\,n}=\frac{\sum_{k=1}^{T}p\,\left(X_{k},Y_{k}\right)*X_{k}}{\sum_{m}^{1}p\,\left(X_{k},Y_{k}\right)}, \end{array}$$

(4) $$\begin{array}{c} Y_{c\,e\,n}=\frac{\sum_{k=1}^{T}p\,\left(X_{k},Y_{k}\right)*Y_{k}}{\sum_{m}^{1}p\,\left(X_{k},Y_{k}\right)}, \end{array}$$

where *p*(*X*_*k*_,*Y*_*k*_) denotes the pressure value of the pressure sensor k of the smart insole, and (*X*_*k*_,*Y*_*k*_) denotes the coordinates of the pressure sensor k in the smart insole coordinate system.

Ultimately, a total of six types of gait features were obtained from the data collected by the wearable device system and the active joint mobility measured clinically, which were subsequently used as input features for the dataset to train different regression models. Because of the patient’s walking ability, the length of collection time and number of collected steps varied. The number of steps collected by each person is between 50 and 150. The data uses one gait cycle as a sample, with a total of 2,352 samples. There were 760 samples in Healthy, 521 in B-Vn 726 in B-IV, and 345 in B-III. The dataset’s dimension after feature extraction is 2352×130.

### Feature analysis and selection

Since the number of features obtained from the current calculation is large, the number of input features needs to be further streamlined using data analysis techniques. In this regard, the relevance and importance of features are further considered in the dimensionality reduction process. Eliminating redundant features not only speeds up the model training process, but also avoids prediction errors due to multicollinearity as much as possible. In this paper, the Pearson correlation matrix between features is constructed by calculating the Pearson coefficients between each feature, such as the correlation between feature *a* and feature *b* as shown in Equation (8).


(5) $$\begin{array}{c} r_{a\,b}=\frac{c\,o\,v\,\left(S_{a},S_{b}\right)}{\sigma \,\left(S_{a}\right)\times \sigma \,\left(S_{b}\right)} \end{array}$$

where *S*_*a*_ is the sample vector of feature *a*, σ(*S*_*a*_) is the standard deviation of feature *a*, and *cov*(*S*_*a*_,*S*_*b*_) is the sample vector covariance of feature *a* and feature *b*.

After removing some redundant features, the importance of the features also needs to be ranked. Based on the feature importance, more robust features can be selected, improving the generalization ability of the model. In addition, by analyzing the importance of features, it is possible to further understand the importance of relevant features for lower limb evaluation and improve the interpretability of the algorithm, which is an area that has received little attention in other studies.

In this paper, we utilize Random Forest (RF) for feature importance analysis. It is easy to implement, and it has high generalization ability, also it is easy to interpret. Random Forest is a machine learning algorithm whose decision process integrates the classification predictions of multiple decision trees to produce a final result. Specifically, the algorithm first performs random sample sampling in the dataset with put-backs, and then randomly selects *M* features as training inputs to construct decision trees (DT). After the above steps are repeated *K* times, the consequent forest formed by *K* decision trees is obtained. The feature importance evaluation is to evaluate the contribution of each feature to the classification performance of the constructed *K* decision trees, using the out-of-bag (OOB) error rate as an indicator. Specifically, for the kth tree, the number of classification accuracies *acc*_*k*_ is obtained for *n_k_* sample numbers. At this time, the *m^th^* feature *F*_*m*_ is randomly scrambled and a new feature *F*_*m2*_ is formed. After replacing the old feature *F_m_* with the new feature *F*_*m2*_, the *k^th^* tree is retrained and the number of classification accuracies *acc*_*km*2_ is obtained. The classification accuracy is also changed after the replacement of features. By measuring the change of accuracy, the feature importance of the *m^th^* feature *F_m_* for the kth tree *IMP*_*km*_ is:


(6) $$\begin{array}{c} I\,M\,P_{k\,m}=\frac{a\,c\,c_{k}-a\,c\,c_{k\,m\,2}}{n_{k}} \end{array}$$

In this, if there is no feature *F_m_* in the *k^th^* tree, then *IMP*_*km*_ is defined as 0. For a RF of *k* trees, the importance of the feature *F_m_* is:


(7) $$\begin{array}{c} I\,M\,P_{m}=\frac{\sum_{k=1}^{K}I\,M\,P_{k\,m}}{K\cdot \sigma } \end{array}$$

where σ is the standard deviation of *IMP*_*km*_ of *K* tree.

### Model training and evaluation

Our goal is to create a classification model that predicts BRS-L grade from the selected features. In addition to the Random Forest mentioned above, we examined various common effective classifiers, including Naïve Bayes (NB), Support Vector Machine (SVM), k-Nearest Neighbor (kNN). We use cross-validation methods to evaluate the prediction performance of the classification models. In specific, we apply the leave-one-subject-out strategy to divide data into training set and test set. For each iteration, we choose the data of one subject as the test set, and the data of the remaining 29 subjects as the training set. The results of the test sets are aggregated and compared to the entire data set to determine accuracy. And finally, we take the average prediction accuracy as the final result.

In the training process, according to Bayes’ theorem, the probability of occurrence of the corresponding classification under different data features can be known, and the category with the highest probability is finally selected as the final result. SVM makes classification by finding the vector in the data feature space that maximizes the classification interval. In this process, the kernel function plays a crucial role in the performance of the model and needs to be further tuned during the training process. The kNN classifies the data by classifying them under known different categories. When a data point to be predicted appears, it is categorized according to the distance of that data point from other data points already classified, i.e., the closer to which category it is classified.

It should be noted that the model utilized in this paper is a multi-category algorithm model, so the performance of the model needs to be evaluated using the evaluation metrics under multi-category, and the performance measures of the model generally have the following metrics.

The detection rate *P* (Precision) is the proportion of correctly predicted cases to the total sample size. And for the multiclassification problem, can be expressed by the Macro-average method. The recall for a single category is the ratio of positive cases correctly predicted to all positive cases. The F1 value is then summed average of the accuracy rate and the completion rate, which can characterize the importance of the accuracy rate as well as the completion rate (Park et al., 2020).

In addition, the results can be plotted using the subject operating characteristic curve (ROC), which is based on the true case rate as well as the false positive case rate, where the more ideal the classification situation is, the fewer samples are expected to be incorrectly predicted, and the closer the ROC curve should be to the upper left corner. The indicator AUC (Area Under Curve) is defined as the area under the ROC curve, and the closer to 1, the better the model performance.

## Results

### Comparison of feature characteristics

**FIGURE 3:**
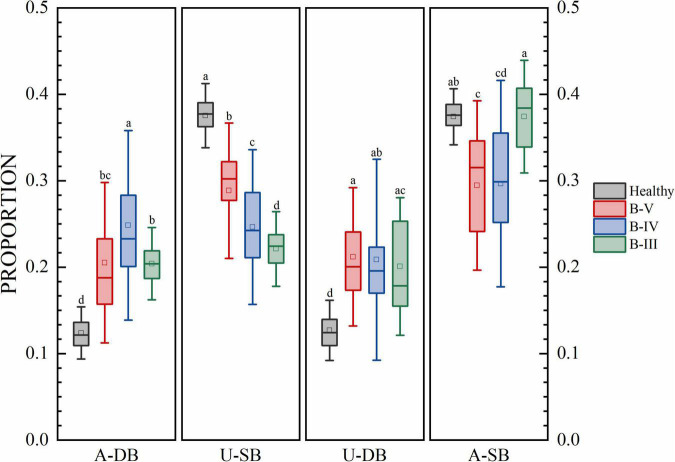
The proportion of each gait phase of subjects at each stage. Different low case letters above columns indicate statistical differences at *P* < 0.05.

We analyzed the difference of gait phase time ratio, knee motion and step length in different hemiplegia grades. The one-way Analysis of Variance (ANOVA) was used, and the homogeneity test of variance had a *p*-value of 0.05, which indicated that variance was not uniform. So, Tamhane’s T2 was used for non-parametric test. The results were show in Figure 3, and the significant differences of characteristics between different groups were marked. Different low case letters above columns indicate statistical differences at *P* < 0.05.

The results for the gait phase parameters are shown in Figure 3 for the proportion of gait phases of four subjects, namely, the affected side double support phase (A-DB), the affected side single support phase (A-SB), the healthy side double support phase (U-DB), and the healthy side single support phase (U-SB). There were significant differences in U-SB among patients with different hemiplegia grades. In A-DB, U-DB, and A-SB, a few groups had no significant differences, but the group with no significant differences did not overlap in the three features. There were significant differences in gait proportion among patients with different grades of hemiplegia. The percentages of healthy individuals in A-DB, A-SB, U-DB, and U-SB are near 12, 38, 12, and 38%. In addition to this, the percentage of bilateral gait phases in healthy individuals with the normal function of both lower limbs in one gait cycle is also symmetrical. In contrast, patients with B-V reached 20% in both dual support phases, i.e., the proportion of time spent in the dual support phase was increased and patients needed to stay on both feet for a longer period. Patients in B-IV stayed in the double support phase for a longer time, while the swing process was shorter on the affected side. For the B-III patients which have more severe conditions, the double support phase was longer in proportion, and symmetry became worse. We can see that the more serious the degree of hemiplegia, the lower the proportion of swing time on the healthy side, because the hemiplegia weakens the supporting ability of the hemiplegia side.

**FIGURE 4:**
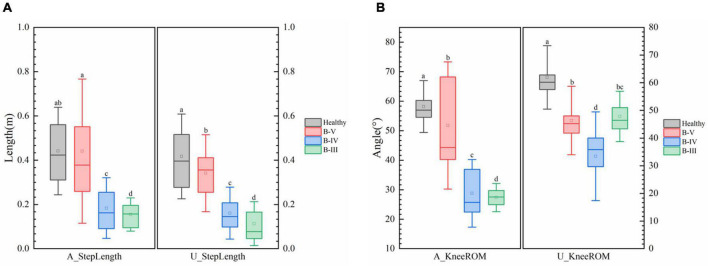
**(A)** Comparison of step length and **(B)** knee range of motion (ROM) in patients with different degrees of hemiplegia. Different low case letters above columns indicate statistical differences at *P* < 0.05.

Figure 4 shows that patients with different grades of hemiplegia showed significant differences in step length and knee motion range. Generally, the knee motion range and step length both reduced as the severity of hemiplegia increased. From B-III to B-IV, although the range of motion of the healthy side of the knee joint was reduced, but the affected side was increased, and the step length was also increased, indicating that the affected side function was initially restored. From B-IV to B-V, the knee motion and step length of the affected and healthy sides were significantly improved. Hospitals also consider B-V to be discharge level. Comparing with healthy subjects, the step length of B-V patients was close to the normal level, and there was also a large range of motion of the knee joint, but the variance of the affected side of the knee joint was large, indicating that the joint stability of the affected side was still poor. According to previous studies on hemiplegic gait, the results of the present study are consistent with the reality that hemiplegic patients have slowed gait speed and compensated for the healthy side in the presence of nerve damage and muscle strength deficit.

### Characterization and model analysis

**FIGURE 5:**
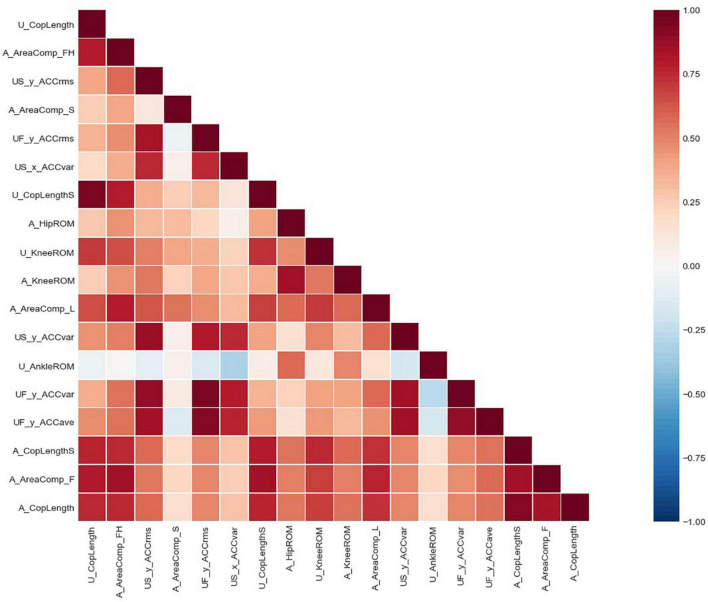
Correlation matrix of top 18 most important features.

**FIGURE 6:**
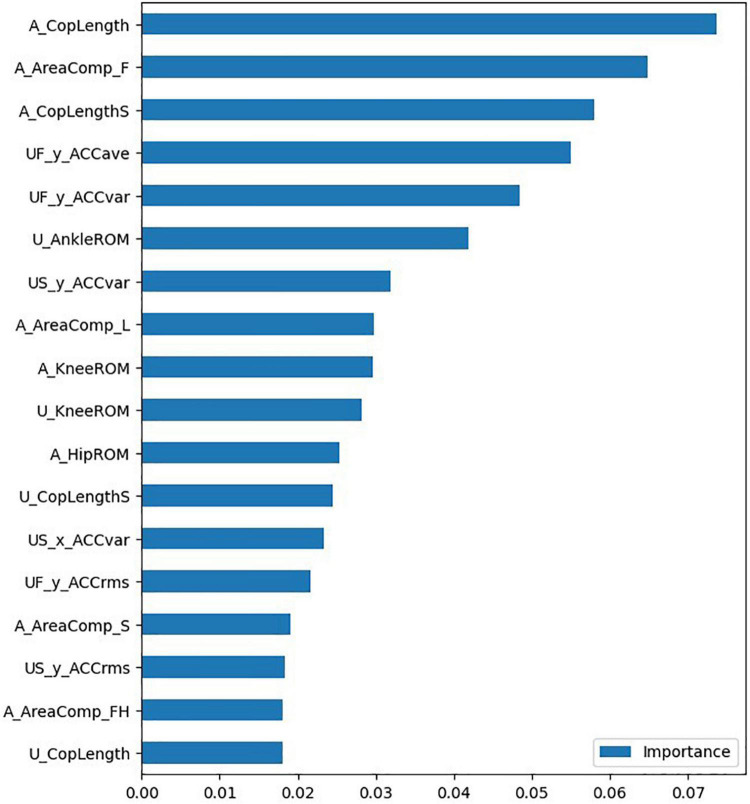
Feature importance ranking (top 18).

The correlation matrix and the degree of redundancy of the data were obtained by correlation analysis of the gait parameters and their characteristics. According to the correlation, features with an absolute value of correlation greater than 0.85 should be excluded. The process of feature rejection also requires the selection of features according to their importance. The correlation matrix of some features is shown in Figure 5. Table 3 and Figure 6 demonstrate the top 18 most important features. The Supplementary material provides the full list of features.

**TABLE 3:** Definition of features in Figure 5.

Feature	Description
A_CopLength	COP trajectory length in affected side
A_AreaComp_F	Forefoot pressure to body weight ratio in affected side
A_CopLengthS	Standard deviation of COP trajectory in affected side
UF_y_ACCave	Average of Y axis acceleration of foot IMU in in unaffected side
UF_y_ACCvar	Variance of Y axis acceleration of foot IMU in unaffected side
U_AnkleROM	Range of motion of ankle joint in unaffected side
US_y_ACCvar	Variance of Y axis acceleration of shank IMU in unaffected side
A_AreaComp_L	Left plantar pressure to body weight ratio in affected side
A_KneeROM	Range of motion of knee joint in affected side
U_KneeROM	Range of motion of knee joint in unaffected side
A_HipROM	Range of motion of hip joint in affected side
U_CopLengthS	Standard deviation of COP trajectory in unaffected side
US_x_ACCvar	Variance of X axis acceleration of shank IMU in unaffected side
UF_y_ACCrms	Root mean square of Y axis acceleration of foot IMU in unaffected side
A_AreaComp_S	Sum of plantar pressure to body weight ratio in affected side
US_y_ACCrms	Root mean square of Y axis acceleration of shank IMU in unaffected side
A_AreaComp_ FH	Hind plantar pressure to body weight ratio in affected side
U_CopLength	COP trajectory length in unaffected side

**FIGURE 7:**
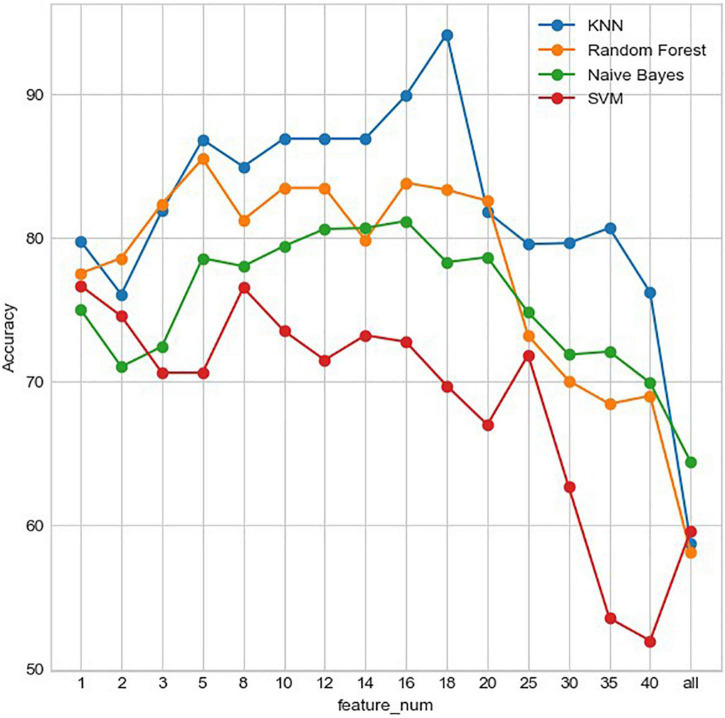
Accuracy of different classification models with the different number of features.

In this paper, the most important features under different numbers are selected for model training based on the importance of the features obtained from the RF algorithm, and the results obtained are shown in Figure 7. It can be seen that as the number of features increases, the classification accuracy of different classification models shows a trend of first increasing and then decreasing. Among them, the kNN algorithm shows a more stable classification accuracy for different numbers of features. When the number of features is 18, the classification accuracy of kNN is the highest, reaching 94.2%. If we use all features as input, i.e., there is no feature extraction, the classification performance of the models will be very poor (accuracy lower than 65%).

Since the highest classification accuracy can be obtained by using the first 18 significant features, the performance of the model under this condition is further analyzed in this paper.

**FIGURE 8:**
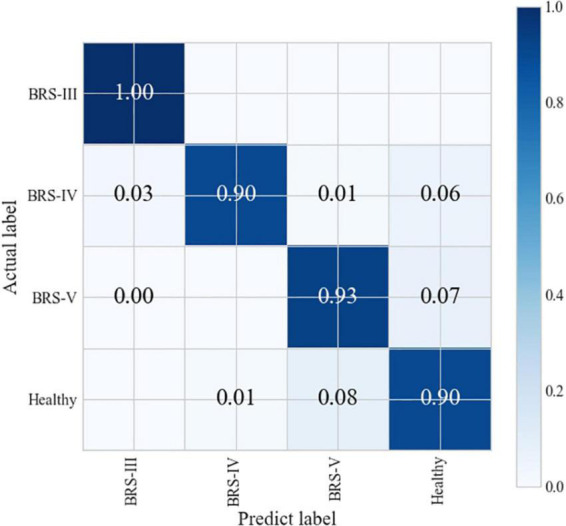
Confusion matrix of the kNN model.

The confusion matrix (Figure 8) shows that the healthy individuals are more accurately classified with B-IV. The major recognition errors mostly occur in patients with B-III incorrectly predicting patients with bit B-IV.

**FIGURE 9:**
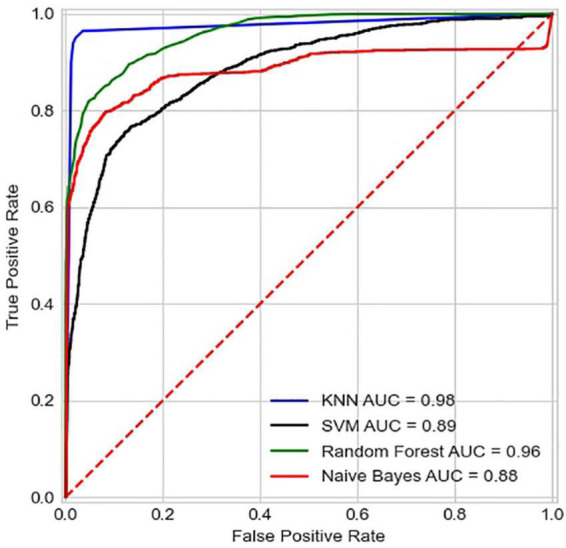
ROC of different classification models.

Comparing the various algorithmic models mentioned above (Table 4), it can be seen that the kNN algorithm has a higher recognition accuracy of 94.2%. The classification accuracy of the other algorithmic models is the lowest at only 69.72%. As can be seen from the Figure 9, the ROC curve of the kNN algorithm is in the upper left corner, and the AUC value at this point is 0.98. Therefore, the most effective and convincing results were achieved by using the kNN model. The results shows that machine learning-based degree evaluation using wearable sensors is feasible and accurate.

**TABLE 4:** Accuracy of classification results.

Model	NB	kNN	SVM	RF
Accuracy	82.43	94.2	75.35	80.07
F1	64.53	93.18	75.56	71.93

## Discussion

This paper proposes an evaluation method based on feature selection and machine learning for automatically assessing BRS-L grade. We extracted a large number of features from the lower limb motion and plantar pressure data collected by wearable sensors. Then we build several machine learning models to classify BRS-L grade using selected features. The kNN achieved the highest prediction accuracy of 94.2%.

We also discovered several interesting findings. One is that the BRS-L is highly correlated with 18 features (Table 3), this indicates that more clinical attention should be paid to these features of the patient.

The key features found in this paper are also of clinical relevance. It is worth noting that the standard deviation of the range of motion of the ankle on the healthy side is greater, as shown in Table 5, the standard deviation of this index reached 7.1° in B-III patients and 5.7° in B-V patients, and the greater standard deviation also indicates that the range of motion of the ankle on the healthy side contains more information. In fact, the B-III grade complained of weakness on the affected side for nearly 50 days and a circle gait during walking. Therefore, when the muscle strength of the affected side is lacking, the lack of strong support of the body will lead to interference of the ankle motion process on the healthy side, creating a more discrete ankle range of motion.

**TABLE 5:** Statistical analysis of ankle range of motion and the trajectory length of CoP.

Indicators	B-III-A	B-III-U	B-V-A	B-V-U
Mean (°)	20.4	28.1	61.4	72.9
Standard deviation	4.1	7.1	5.2	5.7
Mean (mm)	65.1	68.1	79.2	81.3
Standard deviation	11.1	23.1	11.2	16.3

**FIGURE 10:**
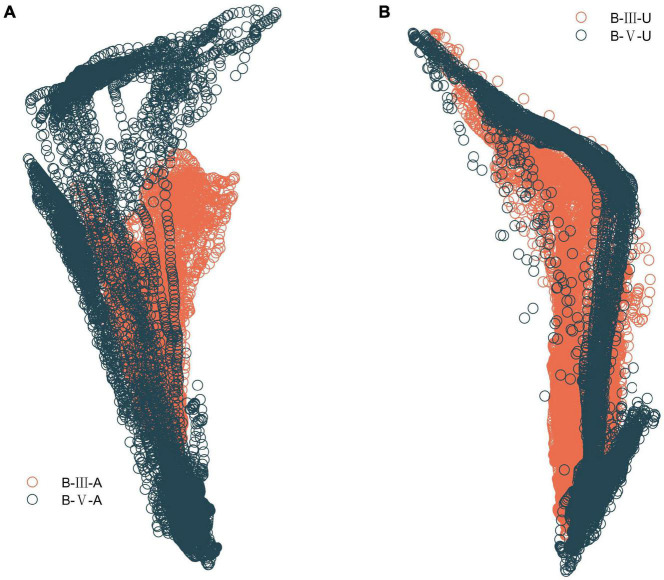
Plantar CoP trajectory in B-III vs. B-V patients. **(A)** Comparison of CoP trajectory on the affected side. **(B)** Comparison of CoP trajectory on the unaffected side.

Similarly, for the first three important characteristics, forefoot pressure values indicate that the patient has weak forefoot stirrups on the affected side, or even does not bring the forefoot to the ground. The standard deviation of the trajectory length of the center of plantar pressure in both feet indicates that the greater the trajectory dispersion, the weaker the patient’s support of the affected side and the less stable the walking. As shown in Table 5, the dispersion on the affected side was greater in B-III patients than in B-V patients. Also, the trajectory length of the center of pressure was shorter in B-III patients, which corroborates with the results in Figure 10, indicating that B-III patients do not have a normal ankle motion process and no forefoot stirrups after heel contact with the ground. For the other important features, also illustrate well their importance for the evaluation, but too many features can lead to a decrease in the classification performance of the model.

Importantly, this paper found that subtle data variation on the robust side was more important for evaluation, possibly because the abnormalities on the affected side were too extreme and varied across individuals, which was detrimental to the generalization of the model. In contrast, the stability of the affected side was better captured by the data variation on the healthy side, and therefore the important features chosen were reasonable.

Since our method is based on gait characteristics, the next step can be to design online correction tools to help patients perform gait correction during the evaluation process (Yang et al., 2018), thus helping patients to consciously perform rehabilitation training, reshape nerves, activate muscles and promote recovery. In addition, by adding visual feedback for stroke patients, patients can visualize their lower limb function and improve the quality of rehabilitation.

## Conclusion

In contrast to previous studies, this paper provides a wearable sensors-based, reliable and interpretable method for evaluating the BRS-L, providing physicians with contextual information for evaluation. Using the dual-source information provided by the wearable device as well as feature analysis, this method is a convenient, accurate, and reliable objective quantitative evaluation method. The accuracy of the feature selection based method in this paper was up to 94.2%. Our method doesn’t require complex setup and thus can provide a home-based evaluation, which greatly reduces the burden on the healthcare system as well as the patient, such as by eliminating the need for frequent trips between home and hospital. Besides, our method greatly improves the relevance and real-time nature of rehabilitation treatment, allowing patients to receive more effective treatment during the prime time of hemiplegia rehabilitation. Because of its simplicity and ease of use, patients can regularly evaluate their stage of recovery, our method is suitable to the home-based rehabilitation.

## Data availability statement

The original contributions presented in this study are included in the article/Supplementary material, further inquiries can be directed to the corresponding author/s.

## Ethics statement

The studies involving human participants were reviewed and approved by the Ethics Committee of the Second Hospital of NanChang University. The patients/participants provided their written informed consent to participate in this study.

## Author contributions

XC and DH designed the research and participated in the entire research including data collection, data processing, model construction, result interpretation, manuscript drafting, and revisions. YZ and JL designed the research and participated in the data collection and revisions of the manuscripts. LX designed the research, manuscript drafting, and revisions. RZ participated in the data collection. ZP and YC participated in the analysis of the results. All authors contributed to the article and approved the submitted version.
