# Supplementary material for: Interpretable evaluation for the Brunnstrom recovery stage of the lower limb based on wearable sensors
Source: Front Neuroinform. 2022 Sep 8;16:1006494. doi: 10.3389/fninf.2022.1006494 (PMC9493089; doi:10.3389/fninf.2022.1006494)
Supplement: Supplementary file 1 [file Table_1.DOCX]

**TABLE 1** **|** Descriptions of IMU Parameters.

| Unit | Accelerometer | Gyroscope | Magnetometer |
| --- | --- | --- | --- |
| Dimensions | 3 axes | 3 axes | 3 axes |
| Dynamic Range | $\pm16g$ | $\pm2000^{\circ}/s$ | $\pm2Gauss$ |
| Sensitivity | $0.0005g/LSB$ | $0.061(^{\circ}/s)/LSB$ | $0.0667mGauss/LSB$ |
| Bias Stability | $\pm20\sim40mg$ | $\pm0.5\sim1^{\circ}/s$ | $-$ |
| Bandwidth | $5\sim256Hz$ | $5\sim256Hz$ | $-$ |
