# Supplementary material for: Interpretable evaluation for the Brunnstrom recovery stage of the lower limb based on wearable sensors
Source: Front Neuroinform. 2022 Sep 8;16:1006494. doi: 10.3389/fninf.2022.1006494 (PMC9493089; doi:10.3389/fninf.2022.1006494)
Supplement: Supplementary file 2 [file Table_2.DOCX]

In the paper, we calculated 130 features from the gait data. Table 1 lists all the features and their definitions. Please note that Table 1 combines some features for brevity, so the number of rows is not 130.

**Table 2: Full list of features**

| Feature | Description |
| --- | --- |
| A_DB | The affected side double support phase time ratio |
| A_SB | The affected side single support phase time ratio |
| U_DB | The unaffected side double support phase time ratio |
| U_SB | The unaffected side double support phase time ratio |
| A_AreaComp_F | Max of the forefoot plantar pressure to body weight ratio in affected side |
| A_AreaComp_M | Max of the midfoot plantar pressure to body weight ratio in affected side |
| A_AreaComp_H | Max of the hindfoot plantar pressure to body weight ratio in affected side |
| A_AreaComp_FH | Max of the front half of the foot plantar pressure to body weight ratio in affected side |
| A_AreaComp_BH | Max of the posterior half of the foot plantar pressure to body weight ratio in affected side |
| A_AreaComp_L | Max of the left plantar pressure to body weight ratio in affected side |
| A_AreaComp_R | Max of the right plantar pressure to body weight ratio in affected side |
| A_AreaComp_S | Sum of plantar pressure to body weight ratio in affected side |
| A_TimeComp_S | Max plantar pressure occurrence time in affected side |
| A_AreaComp_L2R | Max of the left plantar pressure to max of the right plantar pressure ratio in affected side |
| A_SensorComp_12 | Sum of plantar pressure of sensor 1 and sensor 2 to body weight ratio in affected side |
| A_SensorComp_23 | Sum of plantar pressure of sensor 2 and sensor 3 to body weight ratio in affected side |
| A_SensorComp_34 | Sum of plantar pressure of sensor 3 and sensor 4 to body weight ratio in affected side |
| A_SensorComp_45 | Sum of plantar pressure of sensor 4 and sensor 5 to body weight ratio in affected side |
| A_SensorComp_56 | Sum of plantar pressure of sensor 5 and sensor 6 to body weight ratio in affected side |
| A_SensorComp_67 | Sum of plantar pressure of sensor 6 and sensor 7 to body weight ratio in affected side |
| A_SensorComp_78 | Sum of plantar pressure of sensor 7 and sensor 8 to body weight ratio in affected side |
| A_CopLength | Cop trajectory length in affected side (y axis) |
| A_StepLength | Step length of affected side |
| A_CopLengthS | Standard deviation of Cop trajectory in affected side (y axis) |
| A_CopWidth | Cop trajectory width in affected side (x axis) |
| A_CopWidthS | Standard deviation of Cop trajectory in affected side (x axis) |
| U_AreaComp_F | Max of the forefoot plantar pressure to body weight ratio in unaffected side |
| U_AreaComp_M | Max of the midfoot plantar pressure to body weight ratio in unaffected side |
| U_AreaComp_H | Max of the hindfoot plantar pressure to body weight ratio in unaffected side |
| U_AreaComp_FH | Max of the front half of the foot plantar pressure to body weight ratio in unaffected side |
| U_AreaComp_BH | Max of the posterior half of the foot plantar pressure to body weight ratio in unaffected side |
| U_AreaComp_L | Max of the left plantar pressure to body weight ratio in unaffected side |
| U_AreaComp_R | Max of the right plantar pressure to body weight ratio in unaffected side |
| U_AreaComp_S | Sum of plantar pressure to body weight ratio in unaffected side |
| U_AreaComp_L2R | Max of the left plantar pressure to max of the right plantar pressure ratio in unaffected side |
| U_TimeComp_S | Max plantar pressure occurrence time in unaffected side |
| U_SensorComp_12 | Sum of plantar pressure of sensor 1 and sensor 2 to body weight ratio in unaffected side |
| U_SensorComp_23 | Sum of plantar pressure of sensor 2 and sensor 3 to body weight ratio in unaffected side |
| U_SensorComp_34 | Sum of plantar pressure of sensor 3 and sensor 4 to body weight ratio in unaffected side |
| U_SensorComp_45 | Sum of plantar pressure of sensor 4 and sensor 5 to body weight ratio in unaffected side |
| U_SensorComp_56 | Sum of plantar pressure of sensor 5 and sensor 6 to body weight ratio in unaffected side |
| U_SensorComp_67 | Sum of plantar pressure of sensor 6 and sensor 7 to body weight ratio in unaffected side |
| U_SensorComp_78 | Sum of plantar pressure of sensor 7 and sensor 8 to body weight ratio in unaffected side |
| U_CopLength | Cop trajectory length in unaffected side (y axis) |
| U_CopLengthS | Standard deviation of Cop trajectory in unaffected side (y axis) |
| U_CopWidth | Cop trajectory width in unaffected side (x axis) |
| U_CopWidthS | Standard deviation of Cop trajectory in unaffected side (x axis) |
| U_StepLength | Step length of unaffected side |
| A_AnkleROM | Range of motion of ankle joint in affected side |
| A_KneeROM | Range of motion of knee joint in affected side |
| A_HipROM | Range of motion of hip joint in affected side |
| U_AnkleROM | Range of motion of ankle joint in unaffected side |
| U_KneeROM | Range of motion of knee joint in unaffected side |
| U_HipROM | Range of motion of hip joint in unaffected side |
| AreaComp_U2A | Max of the unaffected side plantar pressure to Max of the affected side plantar pressure ratio |
| AF_x(/y/z)_ACCave | Average of X(/Y/Z) axis acceleration of foot IMU in affected side |
| AF_x(/y/z)_ACCvar | Variance of X(/Y/Z) axis acceleration of foot IMU in affected side |
| AF_x(/y/z)_ACCrms | Root mean square of X(/Y/Z) axis acceleration of foot IMU in affected side |
| AF_x(/y/z)_ACCeny | Energy of X(/Y/Z) axis acceleration of foot IMU in in affected side |
| AF_x(/y/z)_ACCabs | Mean absolute value of X(/Y/Z) axis acceleration of foot IMU in affected side |
| AS_x(/y/z)_ACCave | Average of X(/Y/Z) axis acceleration of shank IMU in affected side |
| AS_x(/y/z)_ACCvar | Variance of X(/Y/Z) axis acceleration of shank IMU in affected side |
| AS_x(/y/z)_ACCrms | Root mean square of X(/Y/Z) axis acceleration of shank IMU in in affected side |
| AS_x(/y/z)_ACCeny | Energy of X(/Y/Z) axis acceleration of shank IMU in affected side |
| AS_x(/y/z)_ACCabs | Mean absolute value of X(/Y/Z) axis acceleration of shank IMU in in affected side |
| UF_x(/y/z)_ACCave | Average of X(/Y/Z) axis acceleration of foot IMU in unaffected side |
| UF_x(/y/z)_ACCvar | Variance of X(/Y/Z) axis acceleration of foot IMU in unaffected side |
| UF_x(/y/z)_ACCrms | Root mean square of X(/Y/Z) axis acceleration of foot IMU in unaffected side |
| UF_x(/y/z)_ACCeny | Energy of X(/Y/Z) axis acceleration of foot IMU in unaffected side |
| UF_x(/y/z)_ACCabs | Mean absolute value of X(/Y/Z) axis acceleration of foot IMU in in unaffected side |
| US_x(/y/z)_ACCave | Average of X(/Y/Z) axis acceleration of shank IMU in unaffected side |
| US_x(/y/z)_ACCvar | Variance of X(/Y/Z) axis acceleration of shank IMU in unaffected side |
| US_x(/y/z)_ACCrms | Root mean square of X(/Y/Z) axis acceleration of shank IMU in in unaffected side |
| US_x(/y/z)_ACCeny | Energy of X(/Y/Z) axis acceleration of shank IMU in unaffected side |
| US_x(/y/z)_ACCabs | Mean absolute value of X(/Y/Z) axis acceleration of shank IMU in in unaffected side |
| P_x(/y/z)_ACCave | Average of X(/Y/Z) axis acceleration of pelvis IMU in in unaffected side |
| P_x(/y/z)_ACCvar | Variance of X(/Y/Z) axis acceleration of pelvis IMU in in unaffected side |
| P_x(/y/z)_ACCrms | Root mean square of X(/Y/Z) axis acceleration of pelvis IMU in in unaffected side |
| P_x(/y/z)_ACCeny | Energy of X(/Y/Z) axis acceleration of pelvis IMU in in unaffected side |
| P_x(/y/z)_ACCabs | Mean absolute value of X(/Y/Z) axis acceleration of pelvis IMU in in unaffected side |
