# Supplementary material for: Interpretable evaluation for the Brunnstrom recovery stage of the lower limb based on wearable sensors
Source: Front Neuroinform. 2022 Sep 8;16:1006494. doi: 10.3389/fninf.2022.1006494 (PMC9493089; doi:10.3389/fninf.2022.1006494)
Supplement: Supplementary file 3 [file Table_3.DOCX]

In our paper, we implemented Naïve Bayes, Random Forest, SVM, and kNN using Python and Scikit-Learn. Table 3 lists the key parameters and experimental parameters considered for the work in the Scikit-Learn library of these models.

**Table 3: Parameters and Experimental parameters of machine learning models**

| **Model** | **Parameters** | **Experimental parameters** |
| --- | --- | --- |
| Naïve Bayes | var_smoothing: 1e-9 | -- |
| Random Forest | n_estimators: 50,  criterion: "gini",  max_depth: None,  max_features: sqrt(n_features),  bootstrap: True, | n_estimators: 100,50,20,10,5  max_depth:5,10,20, None (Expand node until all leaves are pure) |
| SVM | penalty: "l2",  kernel: "linear"  loss: "squared_hinge",  Tolerance stopping standard: 1e-4,  Penalty parameter for error items: 0.5,  max_iter: 1000 | kernel: "linear","rbf"  Tolerance stopping standard: 1e-3,1e-4  Penalty parameter for error items: 0.1,0.2,0.5,0.8,1.0, |
| KNN | n_neighbors: 7,  leaf_size: 30,  p: 2,  metric: "minkowski", | n_neighbors: 3,4,5,6,7,8,9,10 |
